# Immune responses to human papillomavirus infection and vaccination

**DOI:** 10.3389/fimmu.2025.1591297

**Published:** 2025-06-16

**Authors:** Eunice Wavinya Kiamba, Martin R. Goodier, Ed Clarke

**Affiliations:** ^1^ Vaccines and Immunity Theme, MRC Unit The Gambia at London School of Hygiene and Tropical Medicine, Banjul, Gambia; ^2^ Department of Clinical Research, London School of Hygiene and Tropical Medicine, London, United Kingdom; ^3^ Department of Infection Biology, London School of Hygiene and Tropical Medicine, London, United Kingdom

**Keywords:** human papillomavirus, infection, vaccination, immune responses, protection, efficacy, therapeutic vaccines

## Abstract

Human papillomavirus (HPV) is the most common sexually transmitted infection. About 90% of HPV infections are transient, resolving without any need for intervention. Most of HPV infections are low-risk non-oncogenic. However, persistent infection with high-risk oncogenic HPV types is the cause of cervical as well as various other anogenital and oropharyngeal cancers. HPV infection on either cutaneous or mucosal surfaces activates both innate and adaptive antiviral immune cells including Langerhans and keratinocyte cells, natural killer cells, B and T cells. These cellular responses alongside their corresponding cytokine profiles have been associated with clearance of HPV infection and regression of HPV associated disease although the actual immune mechanisms involved are not well understood. Current HPV vaccines are based on self -assembled virus-like particles (VLP) from the major viral capsid protein and target the high-risk HPV types as well as two low-risk types responsible for genital warts. The vaccines generate antibody protection against new infections with no effect on already established infections and HPV-associated diseases. Certainly, despite the high effectiveness of current prophylactic HPV vaccines, therapeutic HPV vaccines are needed for treatment of already established HPV infections and disease. Although there have been great efforts in development of therapeutic vaccines, none is yet to be licensed due to low efficacy and safety concerns. There is therefore a need to understand both natural and vaccine-induced immunity, for development of effective and safe therapeutic HPV vaccines. Additionally, a better understanding of the immunogenicity of HPV vaccines, which are among the best subunit vaccines developed to date, may identify immune pathways that could be targeted for development of similarly effective vaccines for other diseases. This review summarises available literature on immune responses to both HPV infection and vaccination, with an aim of improving overall understanding on this subject. This may provide insights for better targeting of both therapeutic and prophylactic vaccines, not only for HPV but also other antigen targets.

## Introduction

Understanding natural immunity to infections may be useful in identification of potential target antigens for precision vaccine development (1). Currently licensed human papillomavirus (HPV) vaccines are highly effective in preventing new HPV infections but do not have effect on those that are already established (2). There is need for better understanding of natural immunity to HPV to aid identify immune molecules and pathways that may be targeted to develop effective therapeutic vaccines. About 90% of HPV infections are transient and clear spontaneously within two years of detection indicating the role of host immunity in viral clearance (3). Additionally, the inverse correlation often reported between regression of HPV associated lesions and functional HPV specific immune cells further indicate antiviral immunity as critical in clearance of pre-cancerous disease (4–6). However, high-risk HPV types apply complex immune evasion mechanisms which enhance their persistence for years and may result to cancer development (7, 8).

Current HPV vaccines generate neutralising HPV specific antibodies that are sustained for years without waning, even after a single vaccination dose (9–11). The mechanisms underlying the production of such long-term antibodies are not well understood with studies on other VLP vaccines including hepatitis B, E and influenza generating less sustained responses (12).

Following a summary of HPV biology and pathogenicity, this review discusses available literature on immune responses to HPV infection and vaccination. Additionally, current progress, gaps and the need for development of effective therapeutic HPV vaccines are highlighted. This comprehensive review contributes to the understanding of immunity against HPV for continued intervention and better control of HPV-associated diseases.

## HPV infection and cervical cancer

Cervical cancer is the most common HPV-related cancer type, ranking fourth among women’s cancers globally and either first or second among cancers affecting women in Sub-Saharan Africa (13). Out of more than 200 known HPV types described and classified into five genera, the alpha genus is responsible for most mucosal and cutaneous infections which are classified into high-risk (oncogenic) or low-risk (non-oncogenic). Each of the high-risk types (HPV 16, 18, 31, 33, 35, 39, 45, 51, 52, 56, 58, 59, 68, 73, 82) has been identified as either single or co-infection with other high-risk types in cervical cancer (14, 15). HPV 16 is the most commonly detected in HPV-related cancers followed by HPV 18, accounting for about 70% of the total, while HPV 31, 33, 45, 52, 58 account for another 19% of cervical cancer cases globally (14, 16).

## HPV biology and pathogenicity

**Figure 1:**
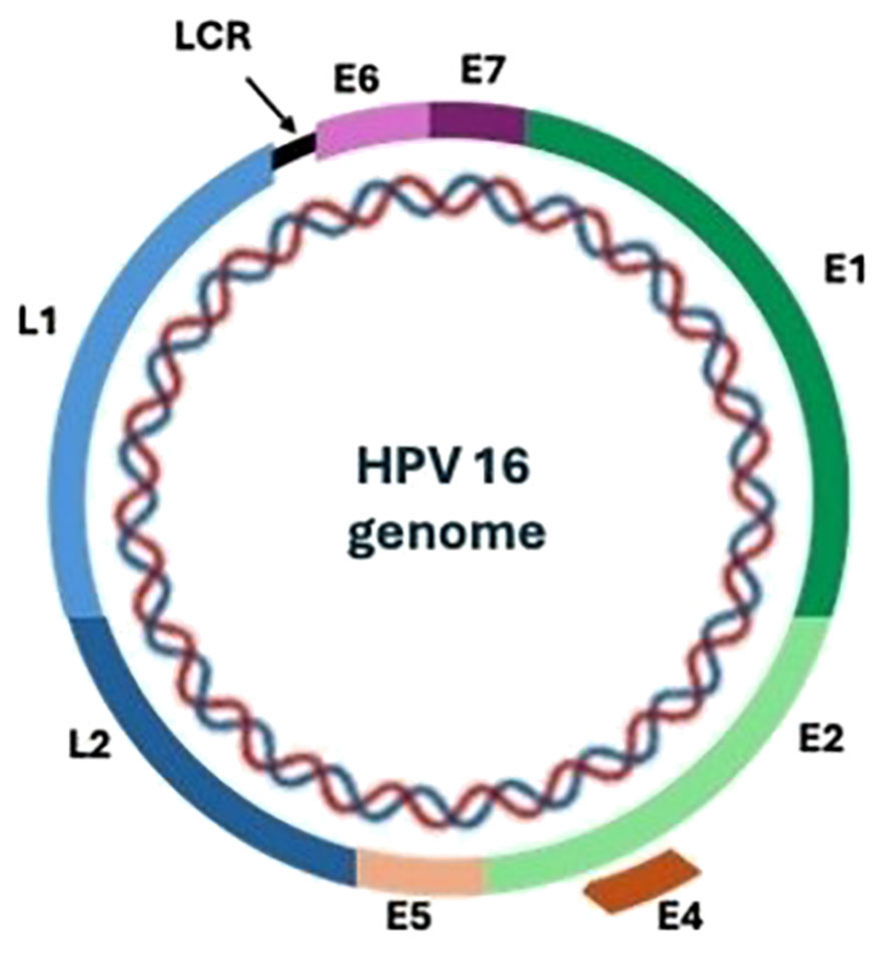
HPV 16 genomic structure. LCR (Long control region): Controls transcription and replication of the viral DNA particularly E6 and E7 expression. Early genes. E1- DNA helicase responsible for recognition of origin for viral genome replication. E2 – Recruits E1 and DNA polymerase to the origin of replication, regulates viral gene transcription. E4 – Plays a role in viral release and transmission. E5 – Interacts with epidermal growth factor and activates immune evasion pathways. E6 – Oncoprotein that interferes with cell cycle by binding the tumour suppressor protein p53. E7 - Oncoprotein that interferes with cell cycle by binding the retinoblastoma gene product pRB. Late genes. L1, L2 – Major and minor viral capsid proteins respectively, assemble into capsomeres.

HPV is a small non-enveloped double-stranded DNA virus, of approximately 55 nanometres in diameter with a complete genome length of about 8 kilobase pairs (17). **Figure 1** shows a general representation of HPV 16 genomic structure. The genome contains a long control region (LCR) and genes encoding six early (E1, E2, E4, E5, E6, E7) and two late (L1, L2) proteins named according to their expression time in the viral life cycle (17).

The early genes are important in viral replication and transcription. They are also involved in dysregulation of host cell processes to enhance viral persistence through interfering with proliferation and differentiation, cell cycle deregulation, controlling cell signalling and inhibiting apoptosis, chromatin remodelling, silencing of tumour suppressor genes and modulation of host immunity and structural modification of the infected cells (18–20). The structural proteins L1 and L2 are important for the viral assembly (17, 21). Protein L1 is the major component of the viral capsid, while L2, the minor capsid protein also plays key roles in the establishment and persistence of infection (17, 21, 22).

Keratinocytes are the main cell type infected by HPV in the cervix (23). At the initial infection stages, in *in-vitro* studies, the viral antigens L1 and L2 are reported to bind to keratinocyte surface receptors or the extracellular matrix and can infect intact cultured epithelial tissues in various epithelial cell lines (22, 24). Such data are contradicted by murine studies which suggest that HPV binds exclusively to the cervical basement membrane which must first be exposed by a microtrauma on the epithelium (24). Several studies reported heparan sulfate proteoglycans (HSPG) as the primary HPV binding receptors on the basal membrane (25–28). This was demonstrated via prevention of HPV infection by N,N′-bisheteryl derivative of dispirotripiperazine or anti-HSPG antibodies that blocked the binding site for possible secondary receptors on cultured human keratinocytes and prevented cell entry (29). Additionally, digestion of cell surface-bound heparan surface (HS) with heparinase I has been shown to suppress pseudo infection of HPV 16 and 33 on cultured human keratinocytes (27). Chinese hamster ovary cells deficient of HSPG were inefficiently infected by HPV as the virus could not bind stably to their surface (30).

Binding of HSPG both in cell culture and *in vivo* triggers a conformational change on the viral capsid, which exposes the N-terminus of the L2 protein for cleavage by furin or proprotein convertase 5/6 (31, 32). This cleavage exposes a basal keratinocyte secondary receptor binding site on L1 protein and is a critical step for most HPV infection (31). The specific secondary binding receptor for HPV is not known. Some *in vitro* studies suggest a cell surface adhesion molecule, α6-integrin but HPV infection has been shown to take place in cells lacking this receptor (31–33). It therefore remains to be elucidated if there is a universal secondary receptor or whether different HPV types use different mechanisms.

Differences reported in initial HPV binding to host cells between *in vivo* and *in vitro* studies may suggest that different mechanisms are relevant depending on infection context. For example; binding of laminin 5 is important for infection of cultured keratinocytes but less important in HPV attachment and infection in murine genital tract (29, 34). It is important to note that *in vitro* studies lack *in vivo* components such as wound signalling pathways reported to be involved in establishment of HPV infection (35). Therefore confident conclusions on mechanisms of HPV infection and immunity should be based on *in vivo* studies.

## Viral internalisation

HPV infection takes place through micro abrasions on the skin or mucosal surfaces. In HPV types requiring α-integrin in their infection process, this enables it to interact with the protein L1 enabling introduction of the virus into the host cell (36).

A clathrin-mediated receptor endocytosis pathway is implicated in internalisation of most HPV types studied to date including HPV 16 and bovine papillomavirus (37, 38). Alternative pathways such as a caveolae-dependent route or the use of tetraspanin-enriched domains as a platform for viral uptake have been reported for the same or different HPV types (39). Factors such as usage of different pathways by different HPV types, maturation stage and nature of capsid (whether VLP or pseudovirion), different experimental manipulations and study end-points may contribute to differences reported from various studies (33). Therefore, despite the extensive literature on HPV internalization, there is no consensus on a universal viral internalisation pathway for those HPV types studies to date.

Internalised viral particles employ a complex mechanism to gain entry to the host cell nucleus where they can exploit the host machinery for their replication (40). Cell cycle progression is required for the viral genome entry into host cell nucleus during the nuclear membrane breakdown in mitosis (41). Then the viral genome initially replicates slowly and establishes itself in the nucleus by attachment to host cell chromatin and is maintained at constant copy numbers during cell division (41). The viral genome replication is tightly linked to the differentiation of the cervical epithelium (42). Basal cells divide both symmetrically, into the basal layer and asymmetrically to enable epidermal stratification (43, 44). The cells moving up through the epithelium differentiate by acquiring various characteristics until they reach the epithelial surface, from where they are shed in a self-renewing process (43, 44). During the cell differentiation process, host cell transcription factors are produced and interact with the increasing viral LCR. This increases the transcription and translation of early viral proteins (45). As cells mature and move towards the epithelial surface, viral capsid proteins are expressed and virions assembled to produce high copy numbers in the terminally differentiated cells at the uppermost epithelial layer that is shed off (21, 46).

HPV infection does not commonly cause oncogenic transformation. However, a few cases of persistent infection with high-risk HPV types can lead to viral DNA integration into host genome, in a process involving a random breakage between E1 and E2 region and subsequent loss of E2 (47–49). Protein E2 is critical for regulation of the expression and activity of E6 and E7 and therefore, its loss interferes with controlled expression of these proteins and allows favourable amplification throughout the differentiation process (47). The oncogenes E6 and E7 target two host proteins required for cell cycle regulation, the tumour suppressor p53 and the retinoblastoma gene product (pRB) (50). Protein E6 causes degradation of p53 through the ubiquitin-proteasome pathway, interfering with DNA repair, G1 arrest and the apoptosis processes (50). Protein E7 binds to pRB and blocks its interaction with E2F transcription factor 6 (E2F6) (51). Interaction between pRB and E2F6, a transcriptional repressor is a critical regulatory step required to activate E2F6 expression in S phase providing a negative feedback mechanism to slow down progression and exit of S phase when other E2F transcription factors are activated (51). Blocking this step therefore disrupts normal cell cycle exit from S-phase and results in uncontrolled cell proliferation (51–53).

Progression of HPV-associated disease on the cervix takes place in several stages from pre-cancerous to early- and late-stage advanced cancer. These are summarized in **Table 1**.

**Table 1:** Progression of HPV infection to cervical cancer: stages and disease grades.

Stage	Description
HPV Infection	Persistent infection with high-risk HPV types, which can lead to cellular abnormalities. Most infections clear spontaneously, but some persist and progress.
Cervical Intraepithelial Neoplasia (CIN) 1	Low-grade squamous intraepithelial lesion (LSIL); mild dysplasia, often resolves spontaneously.
CIN 2	Moderate dysplasia; higher risk of progression to cancer if untreated.
CIN 3	Severe dysplasia; considered a high-grade squamous intraepithelial lesion (HSIL) and a precancerous condition.
Carcinoma in Situ (CIS)	Full-thickness dysplasia of cervical epithelium but has not yet invaded deeper tissues. Considered a pre-invasive cancer stage.
Early-Stage Cervical Cancer (Stage I & II)	Cancer is confined to the cervix (Stage I) or has spread to the upper vagina and nearby tissues (Stage II). May be treated with surgery and/or radiotherapy.
Advanced Cervical Cancer (Stage III & IV)	Cancer spreads to the pelvic wall, lower vagina, or other organs. Late-stage disease (Stage IV) includes metastasis to distant organs such as the lungs or liver.

Cervical Cancer Stages was originally published by the National Cancer Institute. https://www.cancer.gov/types/cervical/stages

## Immune responses to HPV infection

### Innate immunity

Continuous renewal of the basal epithelial layer leads to the formation of keratinized upper layers through the partial activation of apoptosis by cell degradative mechanisms. These keratinized layers act as a barrier against infections (54). Integrins maintain the integrity of the epidermis by mediating adhesion between the cytoskeleton and extracellular matrix and regulate wound healing processes (55). Early anti-viral immunity is principally mediated by Type I interferons (IFN-I) including IFN-α and IFN-β. These induce antimicrobial states in infected cells to limit infection spread, moderate secretion of pro-inflammatory cytokines, enhance natural killer cell (NK cell) response and promote antigen presentation to activate adaptive immunity (56). Persistent high-risk HPV infection is made possible by complex immune evasion mechanisms some of which are discussed next. The limited expression of viral proteins in the early infection stages enables HPV to avoid immune recognition and activation of cytotoxic T cells. The confinement of the viral infection to epithelial cells where immune surveillance is less robust further enables evasion of immune recognition. During the infectious cycle, cytolysis is prevented by the viral modulation of cell survival mechanisms via E5 protein and inhibition of apoptosis mechanisms via the oncogenic E6 and E7 proteins (7). The viral replication and assembly occur in cells pre-destined for apoptosis hence the virus does not induce sufficient inflammatory and other danger signals required to prevent viral replication (57). This balanced control of the host immunity enabling survival of infected cells ensures persistent survival of the virus.

Antigen presenting cells (APCs) including dendritic cells (DCs), Langerhans cells (LCs), macrophages and keratinocytes as well as natural killer (NK) cells play a sentinel role recognizing pathogen associated molecular patterns (PAMPs) via specialized receptors, mainly the Toll like receptors (TLRs) family, to promote innate immunity and initiate adaptive immunity (58, 59). Keratinocytes constitute 95% of cervical epithelium, hence are the primary cells infected by HPV at the basal layer (60, 61). They therefore play a key role in the initiation of HPV infection and activation of adaptive immunity as non-professional antigen presenting cells (62). HPV infection activates keratinocytes to synthesize several signalling and regulatory molecules including Type 1 IFN, intercellular adhesion molecule-I (ICAM-I), antimicrobial peptides, pro-inflammatory cytokines, growth factors and chemokines. These molecules enhance activation and recruitment of other immune cells (63–66). Keratinocytes express various extracellular and intracellular TLRs, notably TLR9 critical for recognition of double stranded DNA viruses (67–70). HPV-immortalized human keratinocytes (HaCaT cells) highly express TLR9 and upon activation upregulate the key pro-inflammatory cytokine TNF-α and IL-1 (71). Several studies on cervical and genital HaCaT cells have demonstrated the ability of transforming growth factor-beta (TGF-β), tumor necrosis factor-alpha (TNF-α) and IFN-α to inhibit proliferation of the HPV infected cells and suppress expression of E6 and E7 proteins (72, 73). These cell line studies demonstrated a role of host immunity in controlling HPV infection.

Effective evasion of recognition by innate immunity is thought to be the hallmark of persistent high-risk HPV infections (5). HPV 16 E6 and E7 interact directly with IFN-I pathways, specifically interfering with signalling through IFN-α and IFN-β (74, 75). This impairment of early immune activation may subsequently prevent clearance of the HPV infection.

TLR9 has been reported to induce antiviral immunity upon HPV infection while also being associated with HPV-related cancers. One study used immunohistochemistry to evaluate TLR9 expression in 96 formalin-fixed paraffin-embedded cervical samples from patients with cervical cancer (76). Results showed that TLR9 expression increased gradually with the histopathological grade as follows: chronic cervicitis (2/17, 11.8%) < low grade cervical intraepithelial neoplasia (CIN 1) (4/19, 28.6%) < medium grade CIN 2 (3/10, 30.0%) < high-grade CIN 3 (12/24, 50.0%) < cervical squamous cell carcinomas (CSCC) (17/32, 53.1%). The authors postulated possible upregulation of unknown TLR9 ligands in the tumour microenvironment resulting to an altered signalling pathway leading to the positive correlation between TLR9 expression and disease severity (76).

A study of 23 patients histologically confirmed to have mild cervical dysplasia, used the polymerase chain reaction (PCR) to determine HPV DNA positivity and persistence over 12 months (77). The activity of NK cells was assessed alongside monitoring of cervical dysplasia by cytology and colposcopy. Immune assessments were performed at diagnosis and after every 3 months over the follow up period. A total of 18/23 (78.3%) of the patients were HPV positive, with high representation of HPV 16 at 55.6% of the total cases. At the end of the study, 12/18 (66.7%) of the HPV DNA-positive women became HPV-negative (defined by at least two negative tests of the original HPV DNA type). In 83.3% of those who cleared HPV, the resolution of HPV infection was associated with clinical pathologic remission of the lesions. Spontaneous and clinical-pathologic remission of cervical dysplasia was associated with higher NK cells activity than persistent disease over the 12 months follow up period (77).

Another study evaluated the immune state in 43 lower genital tract neoplasia patients. Diagnosis was performed by colposcopy with cervical cytology, confirmed by directed biopsy. 21 patients had been affected by recurrent HPV infection either alone or together with intraepithelial neoplasia treated by laser surgery. The other 22 had previously been treated, clinically cured, and did not relapse over 18 - 24 months follow-up. In the patients with recurrent infection, results showed a positive relationship between reduction of NK cells and HPV infection associated with intraepithelial neoplasia. The authors concluded that viral control in the patients who did not relapse is from NK cells targeted to the viral antigens, which may have been suppressed by the persistent infection in the other group leading to the development of lesions (78). An earlier study assessed cytotoxic activity of NK cells from peripheral blood of patients with different cervical cancer stages. Similar cytotoxic activity of NK cells was reported between patients with localised uterine cervix carcinoma stages (I, II, III and IVa) and healthy women, but was decreased in patients with metastatic stages (IVb) (79). Severity of the late stage disease may have suppressed the NK function.

In a study of 59 cervical cancer and squamous intraepithelial lesions (HSIL) patients, disease-associated HPV types were identified by PCR (80). NK cells were isolated from peripheral blood mononuclear cells (PBMC) and analysed for expression of NK cells activating receptors, NKp30, NKp46, NKG2D, NKp80 and 2B4 by flow cytometry (80). Cytotoxicity of the NK cells against K-562 cells was evaluated using flow cytometry. Healthy HPV negative women with no history of abnormal cytology were used as controls. The results showed decreased expression of NKp30 and NKp46 in cervical cancer and HSIL while NKG2D was downregulated in cervical cancer only. Reduced expression of these receptors correlated with HPV 16 detection, low NK cells cytolytic activity and increased disease severity (80). HPV may have suppressed expression of the activating receptors to evade clearance. A study comparing phenotype and function of NK cells between HIV-positive and HIV-negative women with HPV-associated genital warts reported altered phenotype and reduced function of NK cells from the HIV-positive group. This is a possible reason why HIV positive women are normally less likely to clear genital warts and may develop more severe HPV-associated disease outcomes (81).

Taken together, these studies present NK cells as key players in clearance of HPV infected cells and regression of HPV-induced disease, except in cases where the virus succeeds in suppressing the host immunity. The mechanisms by which this suppression occurs in some individuals and not others are not clear and warrant investigation.

Macrophages are key tissue resident immune cells central in maintaining tissue homeostasis for remodelling, elimination of abnormal cells, phagocytosis and regulation of inflammation via cytokine production (82). The role of macrophages in anti-HPV immunity is not well documented. The first study to investigate the role of tissue macrophages in cervical HPV infection and intraepithelial neoplasia (CIN) used immunocytochemistry (83). Monoclonal antibodies MoAb 3.9, that react with most macrophage populations and MoAb Ell, specific for the C3b receptor, CR1 were used. Cervical biopsy samples from 6 women with HPV infection, 10 with HPV associated CIN and 5 controls with normal cervix were tested. The results showed a small population of MoAb 3.9 positive and only occasional MoAb Ell positive macrophages in the normal cervix. In contrast, there was a significant infiltration of both MoAb 3.9 and MoAb Ell positive macrophages in the epithelium and stroma of HPV infected and biopsies with CIN. The authors postulated macrophages to be the first line of defence against HPV infection, either through a direct anti-viral mechanism or non-specific phagocytosis (83). A recent study used single cell RNA sequencing to assess the role of HPV 16 positive macrophages in cervical cancer prognosis (84). HPV proteins E1, E6 and E7 were found to be expressed in both macrophages and malignant cells. Genes such as Wiskott-Aldrich Syndrome Protein (WASP), IQ Motif Containing B1 (IQCB1), Myosin IF (MYOIF) and PDZ Domain Containing 1 (PDZD1) that favour cervical cancer prognosis were found to be expressed in HPV 16 positive macrophages. The expression of these genes was thought to have been induced by the transcription factors Krüppel-like factors (KLFs) which control metabolic and other cellular mechanisms. Tumour-associated macrophages (TAMs) have been implicated in cancer development, metastasis and angiogenesis (85). In cervical cancer, TAMS display different phenotypes, mainly the M2-like phenotype which inhibits anti-tumour T cell responses, attracts Tregs and secrete immunosuppressive IL-10 and tumour growth factor-beta (86, 87). Like other viruses such as HIV, hepatitis B and C viruses, human cytomegalovirus, and poliovirus, HPV is thought to infect macrophages and suppress their antigen presentation capacity but the underlying mechanisms for this are not well understood (83, 88).

### Adaptive immunity

#### Antigen presentation and costimulation

Expression of major histocompatibility (MHC) and costimulatory molecules by keratinocytes and the cytokine milieu produced by keratinocytes and LCs are critical for T cell activation in the microenvironment of the cervix (89, 90). A study on women with abnormal cervical cytology (CIN) and normal squamous epithelium looked at the antigen presenting environment in terms of the expression of Major Histocompatibility (MHC) class II molecules including Human Leucocyte Antigen (HLA)-DR and HLA-DQ, costimulatory molecules (CD11a/18, CD50, CD54 (or ICAM-1), CD58 and CD86), TNF-α and IL-10 on keratinocytes and LCs (91). On keratinocytes, *de novo* expression of MHC II and CD58 expression were found to positively correlate with CIN (91). On the other hand, LCs did not express any costimulatory molecules in biopsies that were normal or those that had CIN (91). In basal keratinocytes, 100% (12/12) of normal biopsies showed constitutive expression of TNF-α, a strong stimulator of LCs which was lacking in a number of CIN samples including 87% (20/23) of CIN 1 and 67% (12/18) of CIN 2 (91). On the other hand, upregulation of IL-10 was observed in a number of CIN lesions: 52% (12/23) of CIN 1 and 44% (8/18) of CIN 3, but not in normal epithelium, 0% (0/12). Expression of HLA-DR, CD54 and CD58 may indicate immune activation in CIN which may have been suppressed by IL-10 and down regulation of TNF-α (91). The observed restricted expression of costimulation molecules and the nature of the cytokine microenvironment created within the epithelium during HPV-associated disease may limit effective immune responses in some CIN lesions.

Most studies have characterised HPV T cell responses targeted to the HPV oncogenic proteins E6 and E7 following cervical infections. These proteins have been shown to suppress TLR9 expression, reducing recognition and antigen presentation by DCs and macrophages (7). Langerhans cells can access HPV proteins at the epidermis (89). They play an important role in presentation of mainly E6 and E7 HPV antigens to naïve CD4+ T cells supported by production of IL-1α and TNF-α from keratinocytes and IL-1β from LCs themselves (89). The granulocyte-macrophage colony-stimulating factor (GMCSF) from keratinocytes promotes LCs maturation to DCs and enhance antigen presentation (64, 92). A comparison of cytokine secretion by cultured normal human cervical keratinocytes, HPV-immortalized cervical and carcinoma cell lines showed a decrease in IL-1α, TNF-α, IL-1β and GMCSF to be associated with persistent HPV infection (65). The decreased cytokine levels may have limited the antigen presentation environment in cervical lesions (65). Other studies have reported decreased density of LCs in epidermal tissues infected with HPV, postulated to be due to normal egress of activated LCs migrating into draining lymph nodes for antigen presentation to naïve T cells (93).

Calcium-dependent adhesion between keratinocytes and LCs mediated by E-cadherin expression by the LCs is required for retention of LCs in the basal epidermal layers (94, 95). A study examining biopsy samples from patients with cervical lesions reported a reduction in both LC numbers and cell surface E-cadherin expression that was mediated by E6 expression in HPV 16 infected basal keratinocytes (94). This was also reported in another study that showed reduction of LCs and depletion of specific LC subpopulations by HPV infection in intraepithelial tissues (94). This disruption of interaction between keratinocytes and LCs as a first line of defense further contributes to immune evasion allowing persistent viral infection.

#### T cell responses

Despite the described interference with the antigen presenting environment, early studies showed that keratinocytes and LCs can present some HPV E6 and E7 antigens to T cells activating them to mount effective antiviral immunity in immunocompetent individuals (96). Recent studies utilizing tetramer-based single-cell and proteome-wide analyses identified E1, E2, E4, E5 and L1 specific T cells in HPV 16- and HPV 33- associated oropharyngeal cancers (97, 98). Investigating HPV specific T cell responses to these proteins following cervical HPV infections may identify potential targets in the development of anti-HPV therapeutics.

One study monitored the regression of HPV 6- and 11-associated genital warts and changes in the immune responses. Regressing warts (n=14) contained significantly more, predominantly CD4+ T cell lymphocytes than non-regressing controls (n=14). Majority of the T lymphocytes in regressing warts were antigen experienced with greater expression of activation markers HLA-DR and ICAM-1 on keratinocytes, and E-selectin and vascular cell adhesion molecule 1 (VCAM-1) on endothelial cells (96). Other studies reported that during HPV 16 infection, Th1 cytokines including IL-2, IL-12 and IFN-*γ* are involved in CD8+ T cell activation and correlate with clearance of cervical lesions expressing HPV E6 and E7 antigens (99, 100). A study investigated the presence and quality of anti-HPV 18 E6 IFN-γ CD4+ T cell responses in the blood of 37 patients with CIN, 25 normal donors, and 20 cord blood samples and compared the responses with clinical outcome (101). Considering subjects with CD4+ T specific reactivity as responders by proliferation and/or cytokine release assays using one or more HPV 18 E6 peptides, the study reported the number of responders to be 0% (0/20) in cord blood samples, 12% (3/25) in normal donors and almost 40% in HPV 18-positive patients depending on the effector function tested. When only HPV 18-positive patients were considered, the responders’ percentage was almost 80%. Since only about 20% of cervical lesions have been reported to be caused by HPV 18 from previous studies, the authors hypothesised that HPV 18 infects a higher percentage and that some of the E6 responsive HPV 18 negative patients may have cleared the infection and developed CD4+ T cell memory. 13 of the responders were followed up after surgery and evaluated for clinical outcome. The initial follow-up times included 3, 6, 9, and 12 months and thereafter every 6 to 8 months, and consisted of clinical inspection, cytology, colposcopy and HPV detection, depending on suspected relapse. At the end of follow up, 62% (8/13) of them had a favourable outcome, defined as being negative for both cytology and colposcopy while 38% (5/13) had unfavourable outcome, defined as being positive for either cytology, colposcopy or HPV detection.

In another study, HPV 18 E7 specific CD4+ T cell responses were evaluated before treatment in patients with CIN or low-grade invasive cervical cancer, and in age-matched healthy controls (102). Upon *in vitro* stimulation of whole peripheral blood mononuclear cells with E7 peptides, the study reported robust Th1 (IFN-γ) and Th2 (IL-4 and either IL-5 or IL-10 or both) cytokine production from HPV 18 negative patients. In contrast, HPV 18 positive patients showed little or no cytokine production (102). Similar analysis for the same Th responses targeted to E6 peptides showed no difference between the HPV 18 negative and HPV 18 positive patients (102). Twenty percent of the healthy controls had E7 specific Th1 and Th2 cytokine production similar to those observed in the HPV 18 negative patients. 16% of the same Th responses targeted to E6 had been reported in the healthy controls (101, 102). The authors concluded that E7-targeted CD4+ T cell response may have cleared and protected against subsequent HPV 18 infection. They note that lack of CD4+ T cell response in the patients with HPV 18 infection may have been caused by successful immune suppression by the infection.

Immunocompetence is key in prevention of persistent HPV infection. Sexually active women living with HIV are more likely to acquire and less likely to clear HPV infection, thus increasing their risk of developing cervical cancer (103, 104). Indeed, 28 000 out of the 33 000 global new cervical cancer cases diagnosed in 2018 were attributed to HIV infection (105). Suppression of CD4+ T cell response by HIV favours HPV infection and persistence (103). Less than 200 CD4+ T cell count per mm^3^ or HIV RNA level of more than 100,000 copies per mL were reported to increase the risk of incident cervical HPV detection and the prevalence of LSIL (104). A nested case control study within a Swiss HIV cohort reported a significant association between the risk of developing CIN 2/3 with low CD4+ T cell counts. The odds of developing CIN 2/3 increased with a decrease in CD4+ T cells (Odds ratio 1.15) per 100 μl/mL decrease in CD4+ T-cells (103). One study assessed long-term (8 years) cumulative detection of HPV among HIV-seropositive women (n=2543) and reported an increase (58% at baseline to 92% at 8 years) in HIV-seropositive women compared to (22% at baseline to 66% at 8 years) in HIV-seronegative women (n=895) (106).

Collectively, CD4+ T cells appear to play a central role in HPV clearance and the impairment of their activity by immunosuppressive strategies of HPV immune evasion or by other infections such as HIV increases the risk of persistent HPV infection and progression to disease.

Like NK cells, CD8+ cytotoxic T lymphocytes (CTL), hold cytotoxic granules required for clearance of virally infected and cancerous cells, hence are important immunotherapeutic targets for cancer treatment (107, 108). Murine immunization with non-tumour fibroblast-like cells transfected with HPV 16 E6 and E7 gene generated CTL anti-tumour response that subsequently inhibited the growth of HPV 16 E6 and E7 positive tumour cells transplanted into them (109, 110). HPV 16 specific CTL responses, defined by the expression of granzyme B and T-cell intracellular antigen 1 (TIA-1) were assessed in 24 randomly selected CIN lesions of increasing severity and in 14 cervical squamous cell carcinomas (111). The samples were also analysed for MHC I expression, often reported to be down-regulated in HPV-induced lesions. Following immunohistochemical analysis, invasive carcinoma had higher expression of CD8+ T cells, TIA-1 and Granzyme B than premalignant CIN 1-3. Substantial infiltration of CTL was observed in CIN in the dysplastic epithelium with about 50% expression of TIA-1, but few Granzyme B lymphocytes were observed in both stroma and neoplastic areas. The reduced Granzyme B in infiltrating CTL may indicate that only a minority of them may have cytotoxic potential, hence it is important to also consider the functional characteristics of infiltrating immune cells carefully. Granzyme B expression in carcinoma samples varied from few (below 5) to massive (over 40) per high-power field. The mean number of TIA-1-positive cells was similar to that of Granzyme B-positive cells in carcinomas. There was no correlation between the overall percentage of activated CTL and MHC I expression, except only when they were infiltrating, which aligns with the CTL role in killing virally infected or tumor cells. Results from this study suggested proper activation of CTL in some carcinomas with their activity likely hampered by local factors or immunoselection of resistant neoplastic cells that inhibit a proper CTL to the neoplastic cells.

The populations of tissue infiltrating immune cells were assessed in a cross-sectional cohort of different CIN grades and a longitudinal cohort of regressing, persistent and progressing CIN 1 (112). At recruitment, 59% (74/125), 31% (39/125) and 10% (12/125) women had histologically proven CIN 1, CIN 2/3 and normal biopsies, respectively. Within one year, 24.6% (17/64) CIN 1 progressed to CIN 2/3. Cytotoxic lymphocytes were the predominant intraepithelial cell population in CIN, while CD4+ and FOXP3+ regulatory T cells predominated the stromal compartment. There were significantly higher number of granzyme B-positive CTL in the entry samples of women with CIN 1 who later regressed, with the ratio between all infiltrating CTL and granzyme B+ cells being close to 1, suggesting high activity. However, this ratio was three-fold lower in women whose lesions persisted or progressed. This suggests that early infiltration of highly cytotoxic effector cells into lesions may protect against disease progression. HPV 16 specific CTL with an ability to lyse HPV 16 infected cells are often reported in tumours and lymph nodes of cervical cancer patients as well as in HPV infections that have not progressed to lesions (100, 113–115). Some studies suggest that E6 targeted CTL may be more important for HPV 16 clearance than E7-targeted CTL (116). Greater loss of MHC I expression has been reported in cervical biopsies with CIN and cutaneous warts compared to condylomas and laryngeal papillomas (117, 118). Other studies have shown a positive correlation between MHC I loss with increased HPV-related disease invasiveness further supporting the immune suppressive effects of HPV in promoting disease progression (119, 120).

While CTL are critical for clearance of HPV infected cells, the mechanisms that determine their variable activity between individuals are not understood. These may be influenced by the HPV infection and disease microenvironment as well as other host factors.

#### Humoral responses

Most literature on HPV humoral immunity at the cervix is limited to HPV 16 and shows infection-induced HPV specific antibodies to be predominantly targeted to L1 and L2 capsid proteins, and to a less extent to non-structural disease-associated early proteins E2, E4, E5, E6 and E7 (121, 122). The HPV capsid proteins are not expressed in early infection stages hence do not trigger a humoral response early on (7, 123–125). Various studies have reported antibody responses to HPV infection mostly using VLP-based enzyme-linked immunosorbent assay (ELISA) and multiplex bead-based assays mainly quantifying total HPV-binding antibodies. Pseudovirion-based and secreted alkaline phosphatase neutralization assays are used to determine viral neutralising titres. HPV specific antibodies are protective but the exact titre required to confer protection is not yet known (126).

These assays therefore report HPV seropositivity as the detection of HPV specific antibodies based on lower-detection cut-offs calculated from testing HPV-naïve individuals and HPV-infected or vaccinated individuals (127). The lack of a standardised assay for measurement of HPV specific antibodies presents a challenge on comparability of data between studies.

The duration of HPV infection, whether transient (HPV type infection clearing) or persistent (infection with the same HPV type for over a year) determines the nature of subsequent development of serological response and or development of HPV associated disease. Both IgA and IgG responses are involved in HPV clearance with IgA appearing earlier than IgG and declining faster while IgG persists. IgA is thus thought to be involved in early viral clearance while IgG indicates long-term immunity (128). Several studies have reported HPV type specific IgA, IgG and IgM responses in individuals with HPV infection, intraepithelial lesions and cervical cancer (129–131). Pooled analysis of data from various studies evaluating natural immunity against anogenital HPV infections in male and female subjects was used to demonstrate that naturally acquired anti-HPV antibodies provide modest protection against subsequent cervical infections (132). This analysis involved 24 000 individuals from 18 countries and results showed significant protection against subsequent infection in female subjects with HPV 16 (pooled RR, 0.65; [95% CI 0.50-0.80] and HPV 18 [0.70; 0.43-0.98] which was not observed in male subjects (HPV 16: 1.22; [95% CI 0.6-01.77] (P = 0.05); HPV18: 1.50; [0.46-2.55]; (P = 0.15)) (132).

A longitudinal study assessed the natural history of antibody responses to HPV infection by comparing incident detection and persistence of HPV 6, 16 and 18 DNA in genital mucosa, time to seroconversion and serum IgG persistence (131). Polymerase chain reaction (PCR) was used for HPV DNA detection and ELISA for measurement of serum IgG. The study enrolled 603 young women, the majority (80.8%) of whom reported two or fewer sexual partners at enrolment hence the HPV types detected during follow up were believed to be from the first exposure and not reactivation. Average time interval, between visits was 4.7 ± 0.9 months, and average length of follow-up was 31.3 ± 18.8 months. 42 women in the study had just initiated sexual activity and were used to determine time to seroconversion following incident infection with HPV. Seroconversion for HPV 6 coincided with detection of respective incident HPV DNA unlike for HPV 16 and 18 which was detected between 6 and 18 months after detection of the respective HPV DNA, indicative of the immune suppression by these high-risk HPV types at early infection stages. Though detectable at different times relative to incident HPV DNA detection, the seroconversion rates were similar for the three HPV types; HPV 6, 16 and 18 at 59.5%, 54.1% and 68.8%, respectively (131). Antibody responses to the three HPV types declined after reaching a peak even in continued presence of HPV DNA. HPV 16 antibody response was highest followed by HPV 18 and lastly HPV 6. The majority of women who seroconverted for HPV 16 and 18 remained seropositive throughout the follow up period. For those with more than one follow-up visits after initial seropositivity, 71.4% of 28 women with incident HPV 16, and 78.6% of 14 women with incident HPV 18 infections remained seropositive at all visits. To the contrary, for those with incident HPV 6 infections, only 34.8% of 24 were seropositive during all subsequent visits suggesting HPV 6 antibody responses are less persistent. Some women with persistent HPV DNA did not seroconvert (131). There was a positive correlation between persistence of HPV 16 and 18 and respective antibody titres which was not observed for HPV 6. Women who were HPV 16 and 18 seropositive at least once and became negative for respective HPV DNA in subsequent visits, also became seronegative (131). A similar delay in seroconversion following HPV infection was reported in another study that assessed the relationship between HPV DNA by PCR, and seropositivity by ELISA in 3 anatomical sites (anal canal, genital and oral cavity) in a total of 384 men (133). The seroconversion rates to single or multiple HPV types HPV 6, 11, 16 and 18 varied by anatomic site with 6.3% (24/384), 18.9% (72/384) and 0.0% (0/384) for anal, genital and oral HPV infection, respectively. This study also reported HPV persistence to be a key factor influencing seroconversion (133). Recent pooled data from the unvaccinated control arms of two large phase 3 trials (NCT00122681 and NCT00867464) showed a significant inverse relationship between high levels of HPV 16 and 18 specific serum antibodies and the subsequent risk of incident HPV 16 and 18 infection. These were therefore naturally induced antibodies potentially protecting against new HPV infections. The analyses included 10752 women for HPV 16 (of whom 18% were HPV 16 seropositive at baseline) and 11169 for HPV 18 (of whom 15% were HPV 18 seropositive at baseline). Antibody response, HPV infection and cervical disease were assessed over 4 years. A total of 1534 incident HPV 16 infections and 1607 cervical pre-cancerous disease at different stages were detected over the 4 years follow up period. There was a reduction in detection of new HPV 16 infections by 37%, 12-month persistent infection by 30%, and atypical squamous cells by 43% among women who were HPV 16 seropositive at baseline. All these reductions were associated with increasing antibody quartiles. Additionally, HPV 16-associated CIN 1 and CIN 2 were reduced with increasing HPV 16 antibody levels (CIN 1 RR = 0.09; [95% CI 0.01-0.68] and CIN2 RR = 0.15; [95% CI, 0.02-1.08]) for the 4^th^ quartile compared to seronegative rates at baseline. A total of 1079 incident HPV 18 infections and 733 cervical pre-cancerous disease at different stages were detected over the four years. Detection of new HPV 18 infection was reduced by 17%. No significant association was observed between baseline seropositivity and the risk of HPV 18 infection. On the other hand, the persistent infection was lower in 3^rd^ and 4^th^ quartiles in those that were seronegative at baseline (134). High antibody quartiles decreased the risk of 12-month persistent infection for 3^rd^ and 4^th^ quartiles, respectively. This association was weaker than what was observed for HPV 16 (134). There was a decreased risk of HPV 18 atypical squamous cells with increasing antibody levels for 4^th^ quartile. The study was not powered to assess associations for CIN 1 and CIN 2 between those that were seropositive or seronegative at baseline (134).

HPV infection-induced IgG and IgA responses in cervicovaginal secretions (CVS) are reported to be low, transient and highly variable (135). One longitudinal study used a bead-based assay to assess HPV 16 IgG and IgA antibody response in CVS samples collected at 4 monthly intervals from 20-24-year-old women (n=292) (136). Antibodies IgG, IgA and secretory piece associated antibodies to HPV 16 were detected in 12% (35/292), 6% (18/292) and 8% (23/292), respectively, of the tested samples (136). HPV 16 specific IgG antibodies at the cervix associated strongly with the detection of HPV 16 DNA within the preceding 12 months (odds ratio (OR), 3.3; [95% CI 1.4-7.8]), while secretory IgA associated strongly with detection of squamous intraepithelial lesions within the last 4-8 months (OR, 6.4; [95% CI 1.9-21.8]) (136). Like HPV seroconversion, this study reported several months delay between infection and detection of HPV specific antibodies at the cervix (136). This may partly explain the positive correlation between infection-induced cervical and systemic HPV specific IgG antibodies (137). While infection-induced HPV-specific anti-L1 neutralizing antibodies in circulation are believed to reach the cervical mucosa and provide protection against new infections (138, 139), there is conflicting evidence indicating inconsistent correlation between antibody levels in the genital tract and systemic circulation (137, 140, 141). Therefore, the relationship between HPV infection-induced antibody responses between the two sites is not clear.

HPV type specific IgG response correlate with early infection clearance, with the antibodies believed to neutralise the virus before cell entry since they are unable to clear the virus once integrated to the host genome (121, 122).

Current prophylactic HPV vaccination generates between 10- and 100-fold higher antibody responses than natural infection, with a moderate to strong correlation between vaccine-induced antibodies in CVS and serum, unlike in infection (135, 139, 142, 143). Further, vaccine-induced antibodies strongly neutralize HPV virus with an avidity 3 times higher than those induced by infection (144).

**Figure 2:**
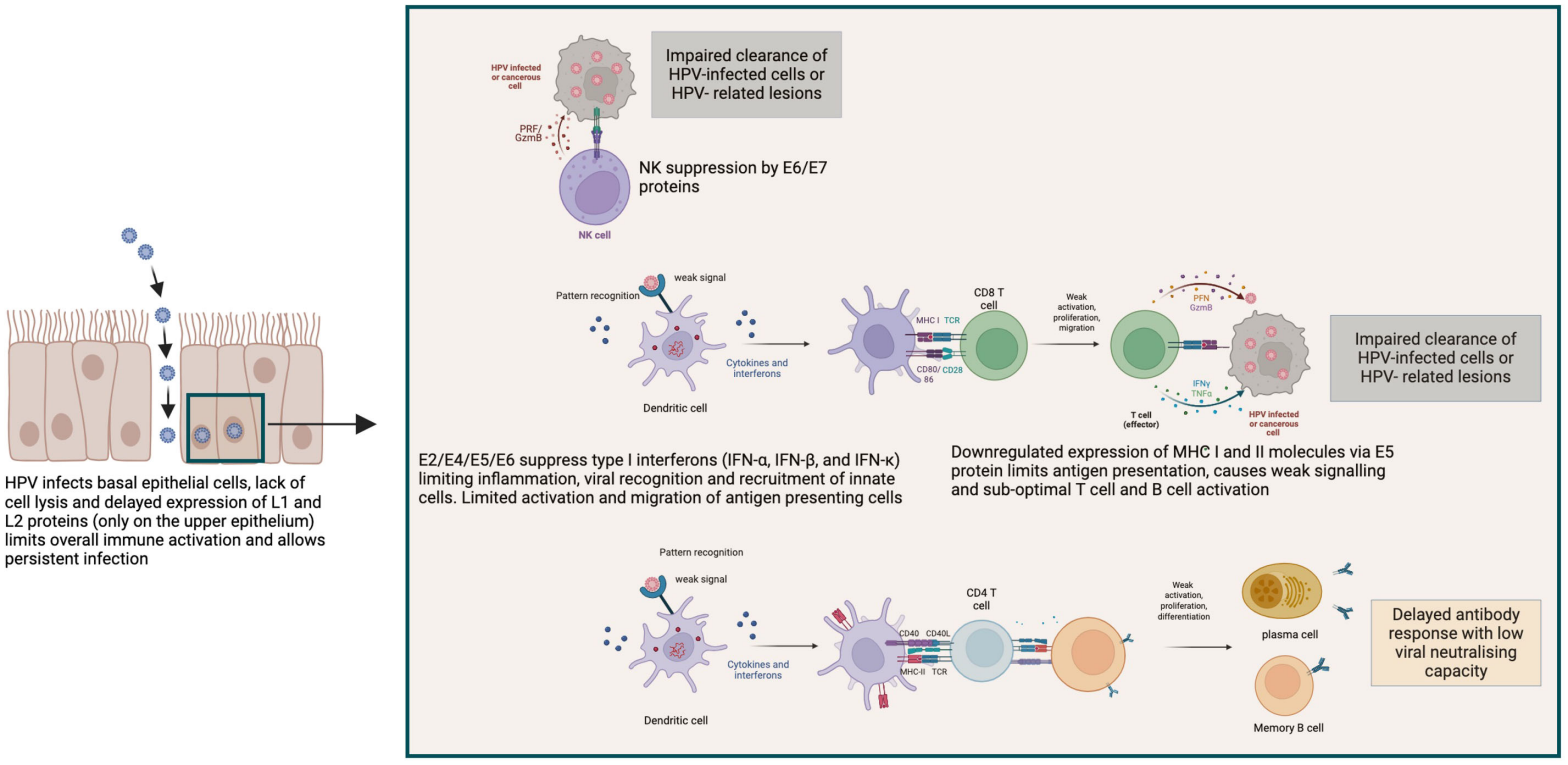
Mechanisms of HPV immune evasion leading to persistent infection and impaired clearance. HPV employs multiple strategies to evade host immune responses, as illustrated in this schematic. HPV infects basal epithelial cells without causing cell lysis or inflammation. The delayed expression of late structural proteins (L1 and L2) until keratinocyte differentiation in the upper epithelium minimizes immune detection and facilitates persistent infection. HPV early proteins (E2, E4, E5, E6, and E7) suppress innate immune signaling by downregulating type I interferon production (IFN-α, IFN-β, and IFN-κ), thereby limiting dendritic cell activation and antigen presentation. The E6/E7 proteins further impair natural killer (NK) cell function, reducing the clearance of infected or transformed cells. E5 inhibits expression of MHC class I and II molecules, leading to reduced CD8^+^ and CD4^+^ T cell activation. Consequently, this dampens the generation of effective cytotoxic responses and limits B cell activation and differentiation. This results to weak and delayed antibody response with low neutralising capacity, contributing to viral persistence and potential progression to HPV-related lesions or malignancies. Created with Biorender.com.

In summary, although HPV infection induces both innate and adaptive immune responses that are able to clear most HPV types, the overall immune activation by high-risk HPV types is very much delayed by poor antigen access and presentation to lymphatics and draining lymph nodes for B and T cell activation (58). The non-cytolytic and non-inflammatory environment renders overall immune activation sub-optimal (**Figure 2**). However, immunocompetent individuals can mount sufficient immune response to prevent persistent infection and enable regression of HPV induced lesions. This demonstrates the critical role of host immunity in controlling HPV infections. Additionally, the high risk of persistent HPV infection and progression to cervical cancer attributable to HIV immunosuppression further demonstrate the critical role of infection-induced host immunity in controlling HPV-associated disease.

## HPV vaccines

There are currently six nationally licensed HPV vaccines, 5 of which are WHO pre-qualified and one under review for WHO pre-qualification (**Table 2**) (145–148). A seventh more recent HPV vaccine has shown non-inferior immunogenicity to the licensed vaccines, with high potential to advance to WHO pre-qualification (148). This development of more HPV vaccines is important in overcoming supply limitations which have been a major challenge in achieving equitable coverage globally.

**Table 2:** HPV vaccines’ information.

Vaccine (licensure year) WHO pre-qualification status)	Valency (VLP types)	Adjuvant	Expression system	Manufacturer
Cervarix (2009) WHO pre-qualified	Bivalent (HPV 16 and 18)	ASO4 (aluminium hydroxide, 3-deacetylated- 4- monophosphoryl lipid A)	*Baculovirus expression vector in Trichoplusi ni* (Hi 5), insect cells	GlaxoSmithKline Biologicals SA
Gardasil (2006) WHO pre-qualified	Quadrivalent (HPV 6, 11, 16 and 18)	AAHS (Amorphous aluminium hydroxyphosphate sulfate)	*Saccharomyces Cerevisiae*, baker’s yeast	Merck Vaccines
Gardasil 9 (2014) WHO pre-qualified	Nonavalent (HPV 6, 11, 16, 18, 31, 33, 45, 52 and 58)	AAHS (Amorphous aluminium hydroxyphosphate sulfate)	*Saccharomyces Cerevisiae*, baker’s yeast	Merck Vaccines
Cecolin (2019) WHO pre-qualified	Bivalent (HPV 16 and 18)	Aluminium hydroxide	*Escherichia coli*, Bacterial cells	Xiamen Innovax Biotech Co. Ltd
Walrinvax (2022) WHO pre-qualified	Bivalent (HPV 16 and 18)	Aluminium phosphate	*Pichia pastoris*	Walvax Biotechnolody Co.Ltd.
Cervavac (2023) submitted to WHO	Quadrivalent (HPV 6, 11, 16 and 18)	Aluminium Hydroxide	*Hansenula*	Serum Institute of India Pvt. Ltd. (SII)

(145–148)

HPV vaccines contain non-infectious VLPs self-assembled from 72 L1 pentameric capsomeres of the HPV types they protect against (145–148).

The HPV vaccination schedule recommended by the WHO was recently updated to one or two doses for females aged 9-20 years and two doses for women older than 21 years. Vaccination primary target is 9-14-year-old girls, with recommendation for secondary targets including boys and older females where feasible (149).

### Vaccine efficacy and immunogenicity

Efficacy of HPV vaccines in humans was first demonstrated against HPV 16 in a randomized controlled trial of HPV 16 vaccine involving 2392 young women (150). Vaccine or placebo was administered in a three-dose regimen (0, 2 and 6 months) and thereafter participants negative for HPV 16 infection at baseline followed up for a median time of 17.4 months. During follow-up, they were tested for persistent HPV 16 infection and CIN at one month after the third dose and every 6 months thereafter. The incidence of persistent HPV 16 infection was found to be 3.8 and 0.0 per 100 women-years at risk in the placebo and vaccinated groups, respectively, translating to 100% efficacy (95% CI 90-100) against new HPV infection (150). Noteworthy, HPV vaccines prevent new infections but do not treat existing infections or diseases (143). Additionally, cross-protection against non-vaccine HPV types has been reported to be mainly driven by HPV 31 and HPV 45 and likely to wane faster than vaccine specific protection (151).

Later studies confirmed this high efficacy against new infections as well as disease caused by HPV types in the vaccine (9, 143, 152–162). When administered to infection-naïve individuals, all HPV vaccines provide close to 100% protection against the HPV types they contain (163). Such high efficacy shown to correlate with the unique induction of exceptionally high antibody levels partly explains why no correlate of protection has been established for HPV vaccines. Serum neutralizing antibodies are important in protecting against new HPV infections but the relationship between their levels and protection has not been verified (164).

Gardasil and Gardasil 9 are similarly efficacious in protecting against the 4 HPV types (HPV 6, 11, 16, 18) they both contain (154).The efficacy of Gardasil 9 was compared to Gardasil, against infection with the 5 additional HPV types (HPV 31, 33, 45, 52, 58) not included in the latter vaccine and development of CIN associated with the HPV types was assessed (154). Gardasil 9 efficacy was 96.7% [95% CI 80.9-99.8] against CIN 2/3, vulvar or vaginal disease associated with the 5 HPV types (154). The efficacy of Gardasil 9 vaccine against infection with all the 9 HPV types it contains, their related diseases and definitive therapy was evaluated in a different (158). Vaccination reduced incidence of CIN 2/3 and cervical surgery related to the 9 HPV types by 98.2% [95% CI 93.6-99.7] and 97.8% [95% CI 93.4-99.4], respectively (158). Similar efficacy was reported for Cervarix vaccine against HPV 16 and 18 infection and HPV 16 and 18-associated CIN 1/2 in individuals vaccinated before exposure to HPV (165). One trial reported Cervarix vaccine efficacy of 100.0% [95% CI 72.3-100.0] against persistent HPV 16 and 18 infection as well as 100.0% [95% CI 61.5-100.0] and 100.0% [95% CI 32.7-100.0] against HPV 16- and 18-associated CIN 1 and CIN 2, respectively (166). Another trial assessing Cervarix efficacy against HPV 16/18-associated CIN reported an efficacy of 96.1% [95% CI 71.6-100.0] and 100.0% [95% CI 74.2-100.0] against CIN 1 and CIN 2, respectively (155).

While there were initially no randomised trials planned to evaluate single dose HPV vaccination, observational data from females who ultimately only received a single dose of either Cervarix or Gardasil vaccines suggested high level protection (11, 167, 168). The Costa Rica HPV Vaccine Trial (NCT00128661) reported Cervarix vaccine efficacy against HPV 16 and 18 infection in 18 to 25-year-old women to be 80.2% [95% CI 70.7 - 87.0] for three-doses, 83.8% [95% CI 19.5 - 99.2] for two-doses and 82.1% [95% CI 40.2 - 97.0] for single dose at 10 years post-vaccination (11). A recent update of this study shows that single dose immunogenicity was still high at 16 years after vaccination with notable declines in the antibody responses between years 11 and 16 (169).

Data from an immunogenicity trial of Gardasil vaccine in India reported a similar ability of a single dose to generate high antibody titres that were stably maintained without waning between 1 and 11 years follow up (168). The study evaluated vaccine immunogenicity in girls aged between 10 and 18 years. The Geometric mean titre (GMT) of HPV 16 at 1 year was 9.72 International Units per mL (IU/mL), 95% (CI 8.30 - 11.37) and at 12 years was 9.90 IU/mL (95% CI 8.76 - 11.19), with similar antibody profiles for HPV 6, 11 and 18 and overall 96% seroconversion (168). Although this single-dose protection has so far been sustained for over 10 years, given the non-randomized nature of these cohorts, hence the inherent risks of bias, single-dose randomised trials have subsequently been judged necessary. Consequently, several randomised trials are ongoing to confirm the single dose vaccine protection. The KEN SHE trial (NCT03675256) of both Gardasil 9 and Cervarix in 3 Kenyan study sites being the first to report high efficacy against HPV 16 and 18 by a single dose of either vaccine given to 15-20-year-old-women (170). Compared to a control group that received meningococcal vaccine, the efficacy of a single dose of Gardasil 9 and Cervarix against both HPV 16 and 18 infection was 97.5% [95% CI 81.7-99.7] and 97.5% [95% CI 81.6-99.7], respectively (170). The ongoing larger ESCUDDO trial (NCT03180034) in Costa Rica aims to compare immunogenicity and efficacy of one and two doses of both Cervarix and Gardasil 9 and is expected to provide definitive outcomes on single dose protection.

An immunobridging analysis comparing the proportions of HPV 16 and 18 seroconverting and IgG antibody geometric mean concentrations (GMCs) between the single dose studies in Kenya (KEN SHE) and Tanzania (DoRIS) two years after vaccination was recently published (171). Findings from this analysis showed that in DoRIS, HPV 16 and 18 antibody GMCs were similar or higher than those in KEN SHE. Cervarix GMC ratios were 0·90 [95% CI 0·72 - 1·14] for HPV 16 and 1·02 (0·78 - 1·33) for HPV 18, while Gardasil 9 GMC ratios were 1·44 [95% CI 1·14 - 1·82] and 1·47 [1·13 - 1·90], respectively. HPV 16 and 18 antibody GMCs and seropositivity from the single dose were non-inferior to two doses for both vaccines. A 5-year follow up of this study has reported durability of single dose anti-HPV 16 and 18 antibodies without waning although non-inferiority of seropositivity from single dose to two doses was maintained for anti-HPV 16 IgG antibodies and not HPV 18 (172).

These data further support the recent recommendation of single dose HPV vaccination, though there is need for data from long-term randomized studies to monitor durability of protection from the single dose schedule. Other ongoing single dose randomised non-inferiority trials include the HANDS HPV vaccine trial (NCT03832049) in the Gambia comparing immunogenicity of one and two doses of Gardasil 9 in girls aged 4-14 years.

Several observational studies have reported a significant reduction in cervical cancer burden and indirect protection at population level in countries with good national coverage of HPV vaccination (173–178). Taken together, these data indicate the effectiveness of HPV vaccines in protecting against infection and disease.

Vaccine-induced IgG antibodies are the mediators of protection against new HPV infections (139, 160). Early animal studies showed immunization with VLPs from papillomaviruses from cottontail rabbits protected domestic rabbits against papillomas caused by cottontail rabbit papillomaviruses (179). Additionally, passive transfer of IgG or serum from immunized rabbits protected against challenge with the same papillomaviruses. This demonstrated antibody mediated protection by VLP-based papillomavirus vaccines (179). Early vaccine studies showed that unlike in murine models, mucosal vaccination via nasal, aerosol or sublingual routes in humans was poorly immunogenic in inducing anti-HPV antibodies in CVS, hence, intramuscular vaccination was studied subsequently (180–182). As a result, HPV vaccines are delivered intramuscularly enabling immediate antigen access to vasculature and lymphatics (57, 183). This induces inflammation with VLP antigens readily accessible to stromal DCs which get strongly activated and migrate transferring the antigens to draining lymph nodes to prime naive B and T cells initiating a strong overall immune activation. HPV vaccine immunogenicity is further thought to be enhanced by mechanisms dependent on the VLP structure as will be discussed later.

The aluminium-based adjuvants used in HPV vaccines enhance the body’s immune response to the VLPs by increasing the local inflammatory response and allowing slow antigen release for sustained immune activation (184). The AS04 adjuvant system used in Cervarix, which is a combination of aluminum hydroxide and monophosphoryl lipid A (MPL), a TLR4 agonist induces stronger innate response responsible for the higher immunogenicity from Cervarix than other HPV vaccines (126).

Unlike the persistent HPV infection that generates both IgG and IgA detectable in CVS, HPV vaccination induces predominantly systemic IgG antibodies (185–187).

HPV vaccine-induced antibody response is broadly documented in systemic circulation but also importantly at the cervix, a major site of HPV infection (135). A number of studies report low levels of detectable vaccine-induced antibodies in CVS that show moderate to strong correlation with the corresponding vaccine-induced antibodies in circulation (135, 185, 188). This is similar to the correlation reported between diphtheria and tetanus toxoid specific antibodies in CVS and serum where the vaccine does not induce antibody response at the genital matrix (185). The antibodies are thus believed to reach the cervix through transudation and exudation (189). Since HPV infects mucosal surfaces, it is important that the vaccine-induced antibodies reach the actual infection site for their protective function. The mechanism of action of HPV antibodies is believed to be viral neutralisation since once an infection has been established, the antibodies are not able clear the virus (126).

Data from randomised trials in different populations demonstrated almost 100% seroconversion to HPV types contained in the vaccine within the first month of vaccination (190, 191). Cross reactivity has also been reported against certain non-vaccine HPV types (192, 193). The antibody profiles from Cervarix, Gardasil and Gardasil 9 show similar patterns peaking one month after completion of vaccination schedule, followed by an initial rapid decline and plateau at around two years onwards with titres well above pre-vaccination (160, 194). Although Cervarix induces higher HPV 16 and 18 antibody titres than Gardasil and Gardasil 9, the high titres from all vaccines are similarly maintained stably without waning (195–198). Available data on the longest follow ups for either of these vaccines show stable antibody persistence for at least 16 years (Cervarix) and 11 years (Gardasil) following vaccination (11, 194, 199, 200).

Majority of data available on vaccine-induced antibody responses in CVS came from Cervarix studies (185, 201, 202). Cervarix vaccination of females aged 14-25 years induced HPV 16 and 18 specific antibodies detectable in CVS (185, 201). Pooled analysis of data from 4 clinical trials involving girls and women reported detectable anti-HPV 16 and 18 antibodies in 95% and 92% of the participants, respectively, 7 months post-vaccination, and ranged between 71-100% and 55-100%, respectively, between 12-36 months post-vaccination (202). Ten years follow-up reported anti-HPV 16 and 18 antibodies detectable in 54-70% and 35-45% of CVS samples respectively, with anti-HPV 16 and 18 antibody titres in serum and CVS showing a mild to strong correlation (correlation coefficients; 0.64 and 0.38), respectively (202). Detection of anti-HPV 16 antibodies remained high (96.3% or higher) in all age groups, while anti-HPV 18 detection was 99.2%, 93.7%, and 83.8%, in 15-25-, 26-45- and 45-55-year olds, respectively (202). Anti-HPV 16 and 18 GMTs were 5.3-fold and 3.1-fold higher than those induced by natural infection, respectively. Persistence of vaccine induced antibodies above infection-induced levels was predicted by modelling to last at least 30 years in all age groups after primary vaccination (202).

### Cellular mechanisms for generation of long-term antibody protection

**Figure 3:**
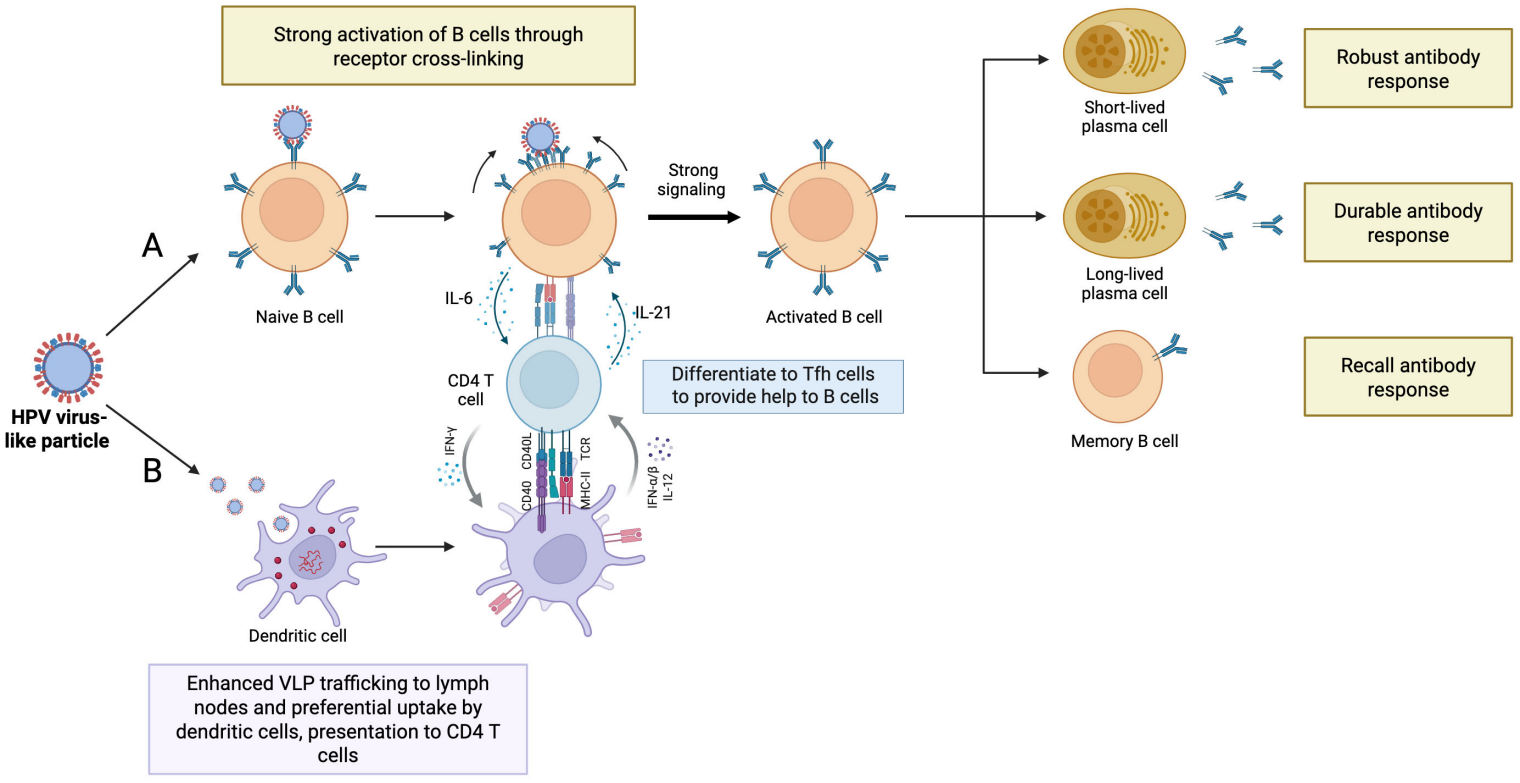
Potent activation of naïve B cells and CD4+ T cells by polymeric HPV virus-like particles (VLPs). Following intramuscular injection, HPV VLPs are trafficked to secondary lymphoid organs where they can activate B cells via two pathways. **(A)** T-independent activation involving B cell receptor cross-linking by repetitive L1 molecules. **(B)** T-dependent activation where the VLPs are preferentially taken up by antigen presenting cells such as dendritic cells which process and present them to naïve CD4+ T cells on MHC II molecules with co-stimulation via other surface molecules such as CD40-CD40L and ICOS-ICOS-L1 and cytokines. The CD4 T cells are activated to differentiate to Tfh cells which are further presented with the HPV antigen by cognate B cells. This interaction between cognate Tfh and B cells takes place in germinal centers. Both pathways can activate B cells to differentiate to short-lived plasma cells for early short-term protection, or to long-lived plasma cells and memory B cells, the sources of long-term antibodies. Tfh, T follicular helper cell; MHC II, major histocompatibility complex II; ICOS, inducible T-cell co-stimulator; ICOS-L1, ICOS Ligand; IL, interleukin; CCR/CXCR chemokine receptors, TCR, T cell receptor; IFN, interferon; IL, Interleukin. Created with Biorender.com.

The immunological mechanisms underlying the nature of long-term antibodies induced even after a single HPV vaccination dose are not well understood. B cell and CD4+ T cell responses are key drivers of long-term antibody production through the germinal center reaction that yields memory B cells (Bmem) and long-lived plasma cells (LLPCs) (**Figure 3**).

Despite the crucial role played by cellular immune responses in determining the nature of antibody durability after vaccination, they are understudied following HPV vaccination. One of the earliest HPV vaccine studies evaluated the innate and adaptive cytokine responses before first dose and one month after second and third vaccination doses with non-adjuvanted HPV 16 L1 vaccine (203). Twenty women received the vaccine and 4 received normal saline as placebo and their whole blood collected before and after vaccination was cultured with HPV 16 L1 VLP stimulation. A multiplex bead-based assay was used to measure the cytokine responses. To compare the cytokine profiles with antibody responses, anti-HPV 16 IgG serology was performed before and after vaccination using ELISA. A broad spectrum of both innate and adaptive cytokines grouped into inflammatory (IL-1β and IL 8), Th1 (IFNγ, TNF-α, IL-12 and GM CSF) and Th2 (IL- 4, IL-5, IL-6 and IL-10) were detected in whole blood at levels clearly discriminating between vaccine and placebo groups. The highest increment in all cytokine responses relative to pre-vaccination levels in vaccinated women was detected in whole blood after the second dose given at two months after the first dose. After the third dose, further non-significant increments were observed for IL-2, TNF-α, GM-CSF, IL-4, IL-10, and GM-CSF. There were no significant differences in the cytokine levels at the different timepoints in the placebo group. The cytokine profiles observed in the vaccine group suggested a recall response to the HPV 16 L1 antigen following secondary vaccination. The vaccine induced median antibody levels at 2560 units at months 2 and 7 at ranges of 640 -10240 at month 2 and 640 - 20480 at month 7. Only one vaccine recipient had detectable anti-HPV 16 L1 antibodies at entry with a titre of 160 units. Median antibody titres at enrolment of the placebo group were 0 (range 0 - 640) and ranged between 0 - 640 and 0 -160 at months 2 and 7, respectively. Correlation analyses showed variable trends between different cytokines and there was surprisingly no significant correlation between cytokine responses and anti-HPV 16 L1 antibodies, which may be partly due to the small sample size used (203).

HPV specific Bmem detectable in circulation after vaccination can mount a fast recall response after secondary vaccination (204–208). HPV 16 specific Bmem elicited by vaccination in female adolescents and young women without pre-existing immunity have been characterised using fluorescently labelled HPV 16 pseudovirus and flow cytometry (205). The identified antigen specific Bmem were further analysed by RNA sequencing, immunoglobulin cloning and assessment of the neutralisation ability of the cloned antibodies. Antibodies cloned from HPV Bmem were mainly of the IgG isotype, followed by IgA and IgM isotypes and used various heavy chain genes (205). Cloned antibodies had a high HPV 16 neutralising capacity *in vitro* despite low levels of somatic hypermutation, normally used as a measure of affinity maturation of antigen specific B cells (205). In another study, high antibody levels from the first HPV vaccination dose were suggested to neutralize the vaccine antigen when administered at short timing intervals (0, 2 and 6 months) compared to a fourth booster dose administered 24 months after the 3-dose schedule (207). Although the delayed fourth dose did not enhance affinity maturation of vaccine specific Bmem, it induced higher antibody titers than the third dose.

Another study reported that a single HPV vaccine dose improved the quality of HPV specific Bmem and boosted antibody titres in previously infected subjects (206). Ten women aged 27-25 years, and with antibodies against HPV 16 were recruited to the study. Five of them received a single dose of Gardasil vaccine and the rest were non-immunized controls. Blood samples were collected 6 months before vaccination, immediately before vaccine administration and later at one week, one month, and 6 months after vaccination. Plasma and peripheral blood mononuclear cells were isolated and used for the measurements of antibody, plasmablast and Bmem responses. HPV 16 specific plasmablasts and Bmem were identified using flow cytometry while antibody responses were measured using both HPV binding and neutralisation assays. HPV 16 specific Bmem were analysed for germline immunoglobulin heavy chain variable gene usage and somatic hypermutation. A robust binding and neutralising antibody response was observed following vaccination. Vaccine-induced antibodies had significantly higher neutralizing capacity than antibodies present before vaccination. Vaccination boosted Bmem numbers 3-27-fold (median 6-fold) and were of the IgG, IgA and IgM isotypes. IgM Bmem were enriched one month after vaccination (206). Only 5 B cell receptor heavy chain genes were identified before vaccination and 3-fold more at one month following vaccination. These data indicate a potential benefit of single-dose HPV vaccination in individuals pre-exposed to HPV.

Whether vaccine-induced HPV Bmem play a role in maintaining long-term antibody protection is unclear. A recent observational study compared antibody and Bmem responses in 149 young adolescents and young adult women at 4-6 years after completion of vaccination schedule with either Cervarix or Gardasil (208). Viral neutralisation and ELISA assays were used to evaluate antibody neutralisation and avidity respectively, while ELISPot was used to measure circulating HPV specific Bmem. With both vaccines, high anti-HPV 16 and 18 antibody responses were sustained through the 4-6 years after vaccination and were higher and more persistent in Cervarix group. HPV 16 and 18 specific Bmem responses generated by both vaccines were similar. The study reported no correlation between vaccine-generated systemic antibody titres and Bmem in either vaccine groups, and concluded that these responses may be independently maintained by mechanisms that remain to be elucidated (208). This absence of a positive relationship between Bmem and antibody responses may indicate that they have little or no contribution in sustaining the long-term antibodies.

Few studies have documented T cell responses to HPV vaccination. An exploratory study on 28 women evaluated T follicular helper (Tfh) cell responses to Gardasil and Cervarix vaccination following a three-dose vaccination schedule (209). Flow cytometry was used to identify activated Tfh cell frequencies ex vivo as CD4+CD45RO+CXCR5+PD-1+ICOS+. Further, the frequencies of Tfh subsets were identified from the total activated Tfh cells based on their expression of the chemokine receptors CXCR3 and CCR6 as Tfh1 (CXCR3+CCR6-), Tfh2 (CXCR3-CCR6-), and Tfh17 (CXCR3-CCR6+). Overall, the results showed higher Tfh1 responses to both vaccines at day 7 post the first dose than post the third dose which is consistent with the earlier discussed data showing high antibody responses after a single HPV vaccination dose. The first Cervarix dose also induced Tfh17 which were not observed in response to Gardasil vaccination, indicating a higher inflammatory response from the ASO4 adjuvant in Cervarix (209).

Another study evaluated the kinetics of innate and adaptive responses generated by Cervarix and Gardasil vaccines (126). Twenty-seven women aged 18-25 years were recruited and randomised to receive 3 doses of either Cervarix or Gardasil. Whole blood samples were collected prior and at different time points after first, second and third vaccination doses. Collected blood was processed into plasma, serum, and peripheral blood mononuclear cells for analysis of different immune responses at different timepoints. For measurement of HPV L1 T cell responses, ELISpot was used to measure IFN-γ responses to L1 peptides. Both CD4+ and CD8+ T cell responses were measured in blood mononuclear cells using flow cytometry intracellular cytokine staining. Serum was used for measurement of HPV 16 and 18 antibody levels and avidity by ELISA, HPV 16, 18, 31, 45 and 58 neutralising antibody titres by pseudovirus neutralisation assays. Increased IL-2 and TNF-α responses were reported in the Cervarix group after completion of vaccination unlike the Gardasil group and was consistent across all 4 tested HPV types (HPV 16 and 18 and cross reactive HPV 33 and 45) (126). Although higher CD4+ T cell responses were achieved with Cervarix after 3 doses, similar affinity maturation was measured for both vaccines. Similarly, despite the higher anti-HPV 16 antibody levels and neutralising titres at month 7, and higher anti-HPV 18 antibody and neutralising titres at months 7 and 12 in the Cervarix group, similar avidity was measured for antibodies from both vaccines. HPV 31 was the only phylogenetically related non-vaccine HPV type tested in this study that was cross neutralised in the Cervarix group (126).

The vaccine-induced cellular differences may explain the differences in antibody titres generated by Cervarix and Gardasil and has been attributed to the different adjuvant systems. While higher CD4+ T cell responses in Cervarix may be expected to enhance affinity maturation than in the Gardasil group, this was not observed. Long-term clinical implications for these differences are unknown but most importantly, both vaccines showed similar avidity, high overall antibody response and have been reported to provide equal protection against HPV 16 and 18 infections (126, 193).

The profile of antibody titres generated by HPV vaccines, plateauing about 2 years post-vaccination and showing no signs of waning or boosting from reactivation of Bmem by viral re-exposure is typically attributable to long-lived plasma cells (LLPCs) (12, 139, 210, 211). However, no studies reported LLPCs from HPV vaccination, potentially due to their homing in the bone marrow. Additionally, murine models do not live long enough and therefore not considered reliable for characterization of LLPCs (12).

Several potential mechanisms have been proposed for immune activation and sustenance of high antibody protection generated by HPV vaccines that is unusual for subunit vaccines (12, 57). The interaction between HPV VLPs with human myeloid antigen presenting cells was investigated to understand how they activate the immune system (212). Monocytes were isolated from PBMC of healthy individuals and were directly stimulated or cultured in appropriate conditions to generate macrophages and DCs. A VLP binding assay utilising a GFP-labelled HPV 16 VLP was used to assess the VLP binding to the cells. HPV 16 VLP was found to bind with an increasing density to the surface of human monocytes, macrophages and monocyte-derived DCs. Production of inflammatory cytokines by the cells was detected using ELISA for TNFα and IL-1β and flow cytometry for IL-1β, IL-12, TNFα and IL-6. The patterns of IL-1β, IL-12, TNFα and IL-6 in response to HPV 16 VLP activation were very distinct from patterns generated by a bacterial activator (lipopolysaccharide), a TLR4 agonist. This targeting of multiple cells, likely facilitated by the vaccine adjuvants was concluded to be one mechanism making HPV VLPs very effective in priming humoral and cellular immunity (212). Antigen size, geometry, kinetics and molecular patterns are important factors in determining the nature of vaccine-induced immunity (213). For HPV vaccines, the polyvalent VLP antigen with particulate 55 nm structure and repetitive array of L1 epitopes on their surface is thought to enhance B cell activation in several ways (212). The small VLP size is efficiently processed by phagocytic cells and traffic to lymph nodes efficiently to activate B cells and T cells (213). The close surface arrangement of the VLP molecules can bind low avidity natural IgM to activate complement, promoting VLP acquisition by follicular DCs (214, 215). The ordered and repetitive L1 antigens are thought to bind strongly to B cell receptors (BCRs) on naïve B cells resulting to BCR-crosslinking through BCR-associated tyrosine kinases. This is believed to generate strong activation and survival signals further alluding to LLPCs as the source of the long-term antibodies (12, 57). Naïve B cells are generally activated by monomeric antigens through IgM and IgD signaling. Repetitive antigens preferentially cause IgD signaling which possibly plays a major role in BCR crosslinking by HPV L1 antigens. Both IgM and IgD signalling are thought to be important in BCR activation by HPV vaccines and warrant further investigations.

## Need for therapeutic HPV vaccines

Current primary vaccination target is younger females to prevent new infections. People with HPV infection and HPV related disease are therefore not considered to benefit from current HPV vaccines except in protecting them from new infections with alternative HPV types. Additionally, cervical cancer patients relapse rates following current standard treatments (chemotherapy, radiotherapy and surgery) increases with the disease stage for example 11-22% and 28-64% relapse rates for stages IB-IIA and IIB-IVA respectively, and different rates in other HPV-related malignancies (216, 217). Persistent HPV infection and disease is more common in immunosuppressed individuals, hence, the therapeutic vaccines in development are targeted to enhance antiviral immunity.

Some of the strategies employed in development of therapeutic HPV vaccines include testing of current prophylactic vaccines as therapeutics for use after treatment, with the aim of preventing disease recurrence and reinfection (218–220). Some studies reported that vaccination with Cervarix or Gardasil after Loop electrosurgical excision procedure reduces HPV disease and infection recurrence (218, 220). However, other studies reported contradicting results highlighting a need for more randomized controlled trials to better understand efficacy of the vaccines in preventing relapses (221).

Various technologies have been explored in the development and delivery of HPV therapeutic vaccines including cell-based approaches, bacterial vectors, viral vectors, peptides or proteins, nucleic acids including (DNA and RNA) (222). Vaccines developed from these approaches have been tested as a single or combined strategies for immunogenicity and effect on cervical pre-cancerous lesions or cancer (222).

DCs are considered ideal immunotherapeutic targets due to their strong ability to initiate and control T cell response. DC-based therapeutic vaccines have been developed in form of DCs pulsed with peptides (or proteins), transduced with either DNA or viral vectors encoding HPV oncogenes E6 or E7 with some failing at pre-clinical phases while others advanced to clinical trials. A placebo controlled phases II and III trial showed no effect of HPV 6 L2-E7 fusion protein on HPV 6-induced warts (223). DC vaccines can induce tumour regression, but are personalised requiring preparation of autologous DCs from individual patients (224). The process is intensive and costly thereby limiting large scale production (224). Additionally, DCs based immunity may not be long lasting since they do not proliferate (225). The use of tumour cell-based vaccines in humans is faced with safety concerns and hence not considered for treatment of HPV-precancerous lesions (226, 227). The earliest protein E6 and E7-based vaccines induced desired immune responses correlating with clearance of HPV infection and regression of HPV-induced lesions but it was unclear if this was due to the vaccine effect or by natural immunity (227).

Commonly used adjuvants, mainly TLR agonists have been tested for their potential in enhancing the immunogenicity of HPV therapeutic vaccines (228–230). The immunogenicity of a therapeutic HPV unadjuvanted vaccine format of synthetic long peptide containing HPV16 E7 antigen, with a centrally located MHC class I epitope was compared to that of the same vaccine adjuvanted with TLR3, TLR4, TLR7/8 and TLR9 agonists in murine models (229). Of these, the TLR9 agonist, CpG oligodeoxynucleotide was the most potent leading to expansion of multifunctional CTL response. Additionally, the vaccine provided both prophylactic and therapeutic benefits. Testing of more recent adjuvants such as α-Galactosylceramide, manganese-doped silica nanoparticles and very small-size proteoliposomes has been proposed as they may potentially enhance the efficacy of HPV therepautic vaccines (230).

Messenger RNA (mRNA)-based HPV therapeutics offer a great promise in targeting E6 and E7 oncoproteins which are consistently present in HPV-associated pre-cancerous and cancerous lesions (231). The efficacy of three different mRNA vaccine modalities in targeting the E7 protein in tumors linked to HPV 16 infection in mice was compared. The modalities included; Self-amplifying mRNA encapsulated in lipid nanoparticles, unmodified and nucleoside-modified non-replicating mRNA vaccines containing a chimeric protein formed by the fusion of the herpes simplex virus type 1 glycoprotein D and the HPV 16 E7 oncoprotein. A single low-dose vaccination with any of the three vaccines activated E7 specific CD8 + T cells, generated T cell memory that could stop tumor relapses, and eliminated subcutaneous tumors at various stages.

Although mRNA-based therapeutics for HPV are promising, their safety remains a major concern, and more research is needed as mRNA technology is relatively at infancy with a lot yet to be understood.

Novel therapeutic approaches continue to be developed for treatment of HPV- associated cancers including combined immunotherapies. A recent phase I/II trial evaluated a combination of tumor-targeting interleukin 12 antibody-drug conjugate (PDS01ADC), the bifunctional anti-programmed cell death ligand 1 (PD-L1)/transforming growth factor β (TGF-β) bintrafusp alfa, and the HPV 16 therapeutic vaccine (PDS0101) for clinical activity in adult patients with HPV 16-associated cancers. The combination of PDS01ADC, PDS0101 and bintrafusp alfa showed an acceptable safety profile and a promising tumor activity as well as improved overall survival of patients with HPV 16-associated cancers (232). These as well as other combination therapeutic approaches warrant further investigations.

Although there has been significant efforts in this area, continued research for better understanding of host immunity as well as exploration of more novel approaches in the development and delivery of HPV therapeutics is needed.

## Conclusion

The host immunity clears most HPV infections through a coordinated response between the innate and adaptive arms, but the underlying immune mechanisms in this process are not well understood, especially in HPV-induced cancerous lesions where the host immunity is extensively modulated by high-risk HPV types. Type I interferons and other cytokines stimulate antiviral immunity via NK cells, keratinocytes and LCs and enhance antigen presentation for clearance of virally infected and cancerous cells by CTLs. Persistent HPV infections and HPV-associated diseases are common in immunocompromised individuals where the virus succeeds to suppress and evade host immunity.

Current HPV vaccines are highly effective in preventing new HPV infections by induction of potent cellular and antibody responses. Whether maintenance of vaccine induced antibodies is dependent on LLPCs and Bmems or both is still unclear. While Bmem have been demonstrated following vaccination, the antibody profile seen at later timepoints following vaccination is more typical of sustained antibody production by LLPCs. More research is required to identify the specific mechanisms or immune pathways activated by HPV vaccination to induce such phenomenal durability of antibodies from VLP antigens. This may help to improve vaccine targeting and may lead to development of other similarly effect vaccines. **Table 3** shows a simplified summary of overall immune responses following HPV infection and vaccination.

**Table 3:** Summary of the nature of immune responses to HPV infection and vaccination.

Aspect	HPV Infection	HPV Vaccination
Antigen exposure	Natural infection through epithelial cells	Viral-like particles (VLPs) in vaccine
Innate response	Weak, as HPV evades detection due to lack of viremia	Stronger due to intramuscular injection and immune activation
Dendritic cells activation	Delayed, as HPV infects keratinocytes without causing inflammation	Rapid activation due to adjuvant presence
B cell activation	Limited; poor antibody response due to immune evasion	Robust; high-affinity antibodies generated
Antibody production	Low, non-neutralizing in most cases	High, long-lasting neutralizing antibodies
Memory B cells	Weak and inconsistent	Strong and long-lived
CD8+ T cell response	Limited due to lack of strong inflammation	Minimal (vaccine primarily induces humoral immunity)
Protection against reinfection	Weak, incomplete	Strong, long-term protection

Despite the limitations encountered in development of therapeutic HPV vaccines, there is hope that continued research for better understanding of host immunity may lead to a breakthrough in this area. Therapeutic HPV vaccines will be vital in controlling infections and preventing advancement to disease in HPV-infected individuals who cannot benefit from current prophylactic vaccines.
